# Comparing PD-L1 with PD-1 antibodies combined with lenvatinib and hepatic arterial infusion chemotherapy for unresectable hepatocellular carcinoma

**DOI:** 10.3389/fimmu.2024.1491857

**Published:** 2024-10-23

**Authors:** Shaohua Li, Jie Mei, Rongce Zhao, Jing Zhou, Qiaoxuan Wang, Lianghe Lu, Jibin Li, Lie Zheng, Wei Wei, Rongping Guo

**Affiliations:** ^1^ Department of Liver Surgery, Sun Yat-sen University Cancer Center, Guangzhou, Guangdong, China; ^2^ State Key Laboratory of Oncology in South China, Guangdong Provincial Clinical Research Center for Cancer, Collaborative Innovation Center for Cancer Medicine, Guangzhou, Guangdong, China; ^3^ Department of Pathology, Sun Yat-sen University Cancer Center, Guangzhou, Guangdong, China; ^4^ Department of Radiation Oncology, Sun Yat-sen University Cancer Center, Guangzhou, Guangdong, China; ^5^ Department of Clinical Research Methodology, Sun Yat-sen University Cancer Center, Guangzhou, Guangdong, China; ^6^ Department of Radiology, Sun Yat-sen University Cancer Center, Guangzhou, Guangdong, China

**Keywords:** hepatocellular carcinoma, PD-1/PD-L1 antibodies, hepatic arterial infusion chemotherapy, lenvatinib, durvalumab, combination therapy, response rate

## Abstract

**Background:**

A combination of hepatic arterial infusion chemotherapy (HAIC), lenvatinib, and immune checkpoint inhibitors (ICIs) yields a high tumor response rate and survival benefit in unresectable hepatocellular carcinoma (uHCC). However, the selection criteria for different ICIs remain unclear. This study aims to compare the efficacy and safety of PD-1/PD-L1 antibodies combined with HAIC and lenvatinib.

**Methods:**

This retrospective study included 184 patients with uHCC treated with HAIC+lenvatinib+PD-1/PD-L1 antibody from June 2019 to January 2022. We utilized propensity score matching (PSM) to select and match 60 patients treated with HAIC + durvalumab + lenvatinib (HDL) against 60 patients treated with HAIC + PD-1 antibodies + lenvatinib (HPL) to compare the efficacy and safety profiles of these two groups.

**Results:**

After PSM, the baseline characteristics were well-balanced between the HDL and HPL groups. The overall survival (p = 0.293) and progression-free survival (p = 0.146) showed no significant difference. The objective response rate (ORR) was higher in the HDL group compared to the HPL group according to modified RECIST (74.1% vs. 53.6%, p = 0.022) and RECIST 1.1 (60.3% vs. 41.1%, p = 0.040), respectively. The incidence of grade 3 or 4 adverse events (AEs) was 10.0% and 18.3% (p = 0.191) in the HDL and HPL groups, respectively.

**Conclusions:**

PD-L1 antibody appears to be a preferable companion in the combination therapy of HAIC + ICIs + lenvatinib compared to PD-1 antibody, showing higher ORR and relatively lower incidence of severe AEs. Further prospective studies involving a larger patient population are warranted.

## Introduction

Hepatocellular carcinoma (HCC) ranks as the sixth most common cancer globally and is the third leading cause of cancer-related deaths (1). In China alone, the burden of liver cancer is significant, with approximately 367.7 thousand new cases and 316.5 thousand related deaths reported in 2022 (2). Unfortunately, the 5-year overall survival rate for liver cancer in China remains low at only 14.1% (3). The onset of HCC is often insidious, and the disease progresses rapidly, frequently leading to diagnosis in advanced stages where curative treatments like resection or transplantation are no longer viable options. For many years, there was a dearth of effective systemic treatments for unresectable HCC (uHCC). However, the landscape has changed dramatically with the rapid development of immune and targeted therapies. Key trials such as Imbrave 150, RESCUE, and ORIENT-32 have demonstrated the efficacy of combining targeted therapies with immunotherapy in significantly improving the prognosis of uHCC patients (4, 5). This paradigm shift has offered new hope for patients previously facing limited treatment options.

Despite advancements in systemic therapies, local therapies such as interventional procedures continue to hold a crucial role in the comprehensive management of liver cancer. Clinicians frequently employ a combination of local and systemic therapies to treat uHCC patients, leveraging the benefits of both approaches. Previous studies have underscored the superiority of hepatic arterial infusion chemotherapy (HAIC) combined with immunotherapy and lenvatinib over systemic immunotherapy combined with lenvatinib (6).

In the phase III HIMALAYA study uHCC, STRIDE (Single Tremelimumab Regular Interval Durvalumab) significantly improved overall survival (OS) versus sorafenib, and durvalumab monotherapy was noninferior to sorafenib for OS (7). The recent HIMALAYA study has further bolstered the arsenal against uHCC, demonstrating positive outcomes, particularly in populations from Hong Kong and Taiwan, with notable long-term survival and high objective response rate (ORR) benefits (8). However, despite these advancements, challenges remain, particularly in the choice of immune checkpoint inhibitor (ICI) regimens. A variety of ICIs are currently available in the clinic, of which the most widely used are PD-1 and PD-L1 inhibitors. Nevertheless, there exists a paucity of robust evidence guiding the selection of different ICI regimens in combination therapy for uHCC. To address this gap, the present study aims to retrospectively analyze the impact of various types of immunotherapies on the prognosis of patients with advanced liver cancer. By elucidating the comparative effectiveness of different ICI regimens, this study seeks to provide valuable insights into optimizing treatment strategies for uHCC.

## Patients and treatment

### Patients

**Figure 1:**
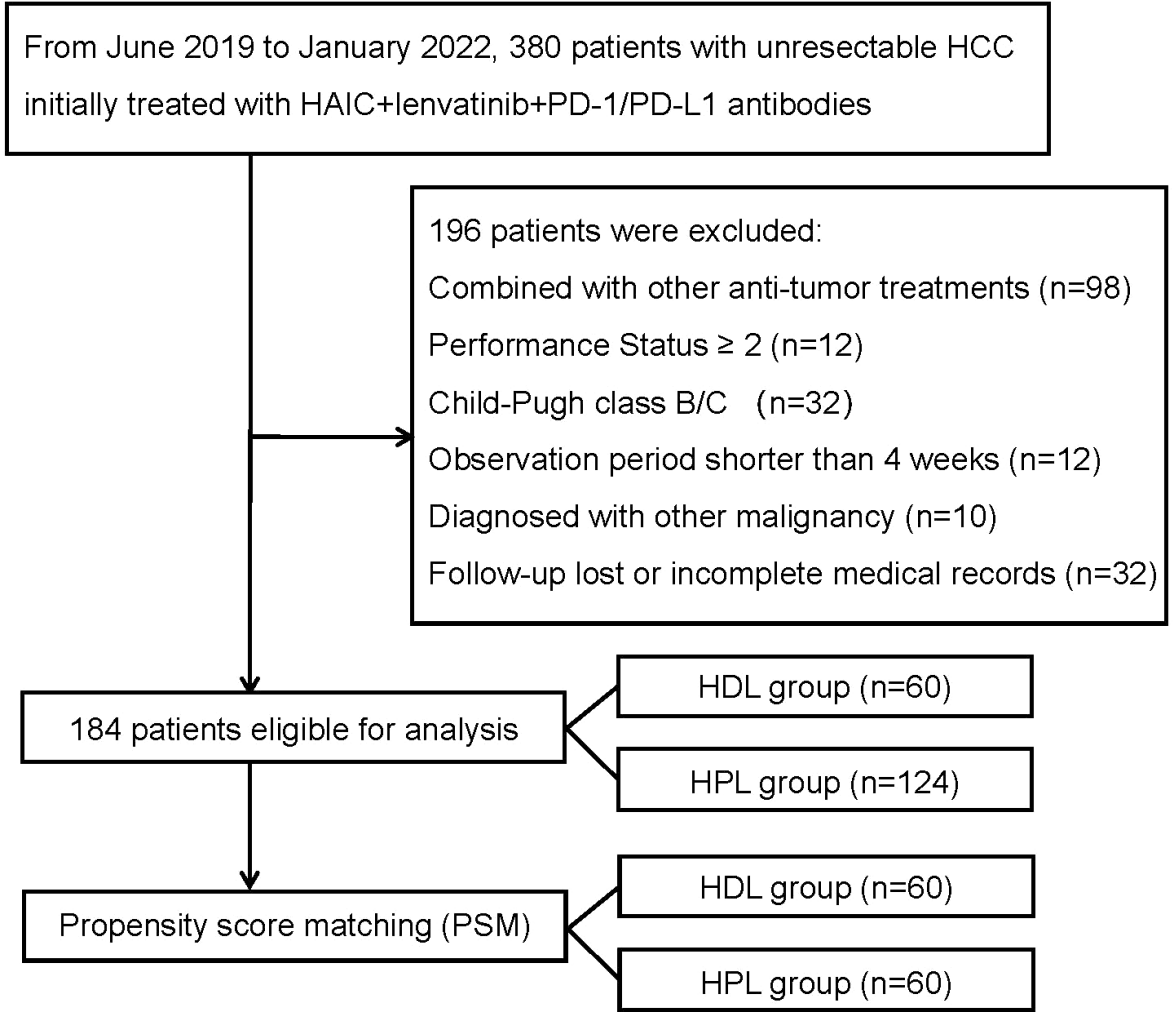
The flowchart of the study. HCC, hepatocellular carcinoma; HAIC, hepatic arterial infusion chemotherapy; HDL, HAIC+durvalumab+lenvatinib; HPL, HAIC+PD-1 antibodies+lenvatinib.

This study was conducted in accordance with the ethical principles outlined in the 1975 Declaration of Helsinki. The analysis of patient data underwent thorough review and received approval from both the Institutional Review Board and Human Ethics Committee at the Sun Yat-sen University Cancer Center (SYSUCC) in Guangzhou, China (Approval Number: B2020-190-01). The study retrospectively included patients diagnosed with uHCC who underwent initial treatment with a combination therapy consisting of HAIC, lenvatinib, and PD-1/PD-L1 antibody from June 2019 to January 2022 at the liver surgery department of Sun Yat-sen University Cancer Center. The inclusion criteria for the study were: (1) Confirmation of HCC diagnosis using either pathological examination or radiological imaging following the Asian Pacific Association for the Study of the Liver (APASL) practice guidelines (9); (2) Unresectable lesions confirmed by multidisciplinary teams; (3) Eastern Cooperative Oncology Group performance status (ECOG PS) of 0-1. (4) Child-Pugh class A liver function; (5) Initial treatment with HAIC + lenvatinib + PD-1/PD-L1 antibody triple therapy. The exclusion criteria were: (1) Patients who received other primary anticancer therapy; (2) Patients who underwent other interventional therapies or received targeted or immune drugs during the triple therapy; (3) Patients diagnosed with other malignant tumors; (4) Observation period less than 4 weeks; (5) Patients with incomplete clinical data or loss to follow-up. The flowchart illustrating the progression of patient selection and inclusion is presumably depicted in **Figure 1**. This rigorous methodology ensures the reliability and validity of the findings while upholding ethical standards in medical research.

### Treatment procedures

The procedure for HAIC described follows established protocols and previous studies (10, 11). Percutaneous hepatic artery puncture and catheterization are performed. Superior mesenteric arteriography and hepatic arteriography are conducted to visualize the blood supply to the tumor. A catheter is then inserted into the blood-supplying artery of the tumor. Patients with an indwelling catheter are shifted to the ward. No implanted port system is applied. The catheter is connected to the injection pump in the ward. Chemotherapeutic drugs are continuously pumped: Oxaliplatin: 135 mg/m^2^ from 0 to 3 hours on day 1. Leucovorin: 400 mg/m^2^ from 3 to 4.5 hours on day 1. Fluorouracil: 400 mg/m^2^ from 4.5 to 6.5 hours on day 1. Fluorouracil: 2400 mg/m^2^ over 46 hours from day 1 to day 3. Patients remain bedridden during chemotherapy. After completion of infusion chemotherapy, PD-1/PD-L1 antibody is injected the next morning and patients are observed for about 2 hours. If no adverse reactions are observed, discharge is arranged. Oral lenvatinib is started on the day of discharge. Dosages of PD-1/PD-L1 antibody and lenvatinib adhere to drug instructions. Patients in treatment cohorts were treated with HAIC + durvalumab + lenvatinib (HDL), while patients in control cohorts were treated with HAIC + PD-1 antibodies + Lenvatinib (HPL). Informed consent is obtained from all patients before treatment initiation.

### Follow-up and assessment

Post-treatment follow-up aligns with routine diagnosis and treatment practices. Reexamination occurs after every two cycles of HAIC. Reexaminations include: enhanced CT/MR of the chest and upper abdomen, electrocardiogram, blood routine, urine routine, biochemistry, coagulation function, tumor markers, etc. Additional examinations such as gastroscopy, thyroid function, and cardiac function if necessary. During treatment, if there’s an opportunity for radical treatment such as surgery, active communication with patients and families occurs, and surgery may be proposed after evaluating the risk/benefit ratio.

Patients received enhanced CT/MR of the upper abdomen within 3 days before the initial of treatment. Tumor response rate included objective response rate (ORR) and disease control rate (DCR). ORR was defined as the percentage of complete response (CR) and partial response (PR) which was maintained for at least 4 weeks from the first radiological confirmation, and DCR was defined as the percentage of patients with CR, PR and stable disease (SD) (12). Tumor response was evaluated according to the modified Response Evaluation Criteria in Solid Tumors (mRECIST) criteria (13) and RECIST 1.1 criteria (14). Adverse events were graded according to the National Cancer Institute Common Terminology Criteria for Adverse Events (NCI-CTCAE) version 5.0. HAIC treatment can be performed up to 8 times. If the patient’s treatment is effective but imaging shows no significant enhancement of the tumor artery, or if liver angiography shows that the tumor has been mostly de-vascularized, HAIC is terminated. Lenvatinib combined with PD-1/PD-L1 maintenance treatment will be used. If the tumor progresses, appropriate follow-up treatment will be decided by the supervising physician based on the individual patient’s condition and response to therapy. In such cases, maintenance treatment with lenvatinib combined with PD-1/PD-L1 inhibitors may be initiated.

### Statistical analysis

The statistical analysis conducted to compare baseline characteristics between the treatment and control groups utilized various tests depending on the nature of the variables. Here’s a breakdown of the methods employed: The distribution of categorical variables was compared using either Pearson’s χ2 test or Fisher’s exact test. For normally distributed continuous variables, the mean and standard deviation were calculated to describe the variable distribution. Student’s t-test was then used to assess the difference in means between the treatment and control groups. For non-normally distributed continuous variables, the median and range were used to describe the variable distribution. The Mann-Whitney test, a non-parametric test, was employed to compare the distributions of these variables between the groups. All statistical analyses were performed using the Statistical Package for the Social Sciences (SPSS) software, version 24.0, developed by SPSS Inc. located in Chicago, IL, USA. A significance level of P < 0.05 (two-tailed) was chosen to determine statistical significance. This threshold indicates that the observed differences between the groups are unlikely to have occurred due to random chance alone.

## Results

### Patient characteristics

This study included a retrospective analysis of 184 cases of uHCC patients who underwent HAIC combined with lenvatinib and either a PD-1 or PD-L1 antibody triple therapy at the Department of Liver Surgery, Sun Yat-sen University Cancer Center, from June 2019 to January 2022. The distribution of patients was 60 cases in the HAIC + lenvatinib + PD-L1 antibody combined therapy group (HDL group) and 124 cases in the HAIC + lenvatinib + PD-1 antibody combined therapy group (HPL group). Notably, all patients in the HDL group received durvalumab (AstraZeneca), while the specific PD-1 antibodies used in the HPL group were detailed in **Supplementary Table 1**. Our analysis revealed significant differences in the proportion of multiple tumors and lesions involving both livers between the HDL and HPL groups (p=0.012 and 0.010, respectively), as indicated in **Table 1**. To address potential biases inherent in retrospective analyses, we employed (PSM) to match and screen selected cases (15). The propensity-score model included gender, serum alpha-fetoprotein (AFP) levels before treatment (≤ or > 400 ng/ml), tumor numbers (single or multiple), liver involvement (unilateral or bilateral), macrovascular invasion (absent or present), and distant metastasis (absent or present). Patients in the HDL and HPL groups were matched in a 1:1 ratio, with a nearest neighbor caliper width of 0.2. This process resulted in a total of 120 patients included in the paired analysis, with 60 patients in each group. Following pairing, the baseline characteristics of the two groups were essentially similar, as summarized in **Table 2**.

**Table 1:** Baseline characteristics of patients in the HDL group and the HPL group.

	HDL group (n=60)	HPL group (n=124)	p value
Age (yr)	51.2 ± 1.5	53.0 ± 1.1	0.321
Gender			0.149
Male,N.(%)	53 (88.3)	117 (94.4)	
Female,N,(%)	7 (11.7)	7 (5.6)	
WBC (×10^9^/L)	6.47 (2.80-12.83)	6.87 (2.57-17.71)	0.066
NE (×10^9^/L)	4.405 (1.11-10.47)	4.40 (1.33-13.69)	0.179
Hgb (g/L)	142.8 ± 2.8	144.0 ± 1.9	0.724
PLT (×10^9^/L)	207 (69-714)	214 (59-662)	0.693
ALT (U/L)	42.45 (0.4-162.1)	48.1 (11.1-251.3)	0.152
ALB (g/L)	42.2 (28.3-51.4)	42.1 (29.6-50.5)	0.647
TBil (umol/L)	14.8 (5.9-55.2)	16.3 (6.0-62.8)	0.193
PT (s)	11.65 (10.2-15.1)	12.0 (9.7-17.1)	0.105
CRE (umol/L)	71.0 ± 1.7	73.2 ± 1.4	0.352
Cycles of HAIC	4 (1-6)	3 (1-7)	0.112
AFP (ng/ml)			0.984
≤400,N.(%)	27 (45.0)	56 (45.2)	
>400,N.(%)	33 (55.0)	68 (54.8)	
HBsAg			0.931
Negative,N.(%)	9 (15.0)	18 (14.5)	
Positive,N.(%)	51 (85.0)	106 (85.5)	
Anti-HCV			0.555
Negative,N.(%)	57 (95.0)	120 (96.8)	
Positive,N.(%)	3 (5.0)	4 (3.2)	
HBV-DNA			0.837
≤1×10^3^ copies,N.(%)	30 (50.0)	60 (48.4)	
>1×10^3^ copies,N.(%)	30 (50.0)	64 (51.6)	
Maximum diameter of tumor (cm)	10.55 (3.4-19.3)	10.0 (1.3-22.1)	0.141
Tumor numbers			0.012
Single,N.(%)	7 (11.7)	35 (28.2)	
Multiple,N.(%)	53 (88.3)	89 (71.8)	
Tumor distribution			0.010
Uni-lobe,N.(%)	18 (30.0)	62 (50.0)	
Bi-lobe,N.(%)	42 (70.0)	62 (50.0)	
Macrovascular invasion			0.955
Absent,N.(%)	23 (38.3)	47 (37.9)	
Present,N.(%)	37 (61.7)	77 (62.1)	
Distant metastasis			0.884
Absent,N.(%)	40 (66.7)	84 (67.7)	
Present,N.(%)	20 (33.3)	40 (32.3)	
Subsequent operation			0.323
Yes,N.(%)	6 (10.0)	19 (15.3)	
No,N.(%)	54 (90.0)	105 (84.7)	

HDL, HAIC+durvalumab+lenvatinib; HPL, HAIC+PD-1 antibodies+lenvatinib; WBC, white blood cell; NE, neutrophil; Hgb, hemoglobin; PLT, platelet; ALB, albumin; ALT, alanine aminotransferase; PT, prothrombin; TBIL, total bilirubin; CRE, Creatinine; AFP, alpha-fetoprotein; HAIC, hepatic arterial infusion chemotherapy.

**Table 2:** Baseline characteristics of patients in the HDL group and the HPL group after PSM.

	HDL group (n=60)	HPL group (n=60)	p value
Age (yr)	51.2 ± 1.5	53.5 ± 1.6	0.303
Gender			0.186
Male	53 (88.3%)	57 (95.0%)	
Female	7 (11.7%)	3 (5.0%)	
WBC (×10^9^/L)	6.47 (2.80-12.83)	6.90 (3.79-17.71)	0.142
NE (×10^9^/L)	4.405 (1.11-10.47)	4.475 (1.70-13.69)	0.352
Hgb (g/L)	142.8 ± 2.8	143.3 ± 2.6	0.907
PLT (×10^9^/L)	207 (69-714)	202.5 (91-662)	0.439
ALT (U/L)	42.45 (0.4-162.1)	49.8 (16.2-209.8)	0.078
ALB (g/L)	42.2 (28.3-51.4)	41.7 (31.0-50.5)	0.723
TBil (umol/L)	14.8 (5.9-55.2)	16.3 (7.0-36.0)	0.099
PT (s)	11.65 (10.2-15.1)	12.3 (10.2-16.3)	0.058
CRE (umol/L)	71.0 ± 1.7	72.2 ± 2.0	0.630
Cycles of HAIC	4 (1-6)	3.5 (1-7)	0.586
AFP (ng/ml)			1.000
≤400	27 (45.0%)	27 (45.0%)	
>400	33 (55.0%)	33 (55.0%)	
HBsAg			1.000
Negative	9 (15.0%)	9 (15.0%)	
Positive	51 (85.0%)	51 (85.0%)	
Anti-HCV			0.619
Negative	57 (95.0%)	59 (98.3%)	
Positive	3 (5.0%)	1 (1.7%)	
HBV-DNA			0.715
≤1×10^3^ copies	30 (50.0%)	32 (53.3%)	
>1×10^3^ copies	30 (50.0%)	28 (46.7%)	
Maximum diameter of tumor (cm)	10.55 (3.4-19.3)	9.95 (2.0-22.1)	0.153
Tumor numbers			0.037
Single	7 (11.7%)	16 (26.7%)	
Multiple	53 (88.3%)	44 (73.3%)	
Tumor distribution			0.130
Uni-lobe	18 (30.0%)	26 (43.3%)	
Bi-lobe	42 (70.0%)	34 (56.7%)	
Macrovascular invasion			0.245
Absent	23 (38.3%)	17 (28.3%)	
Present	37 (61.7%)	43 (71.7%)	
Distant metastasis			1.000
Absent	40 (66.7%)	40 (66.7%)	
Present	20 (33.3%)	20 (33.3%)	
Subsequent operation			0.408
Yes	6 (10.0%)	9 (15.0%)	
No	54 (90.0%)	51 (85.0%)	

PSM, propensity score matching; HDL, HAIC+durvalumab+ lenvatinib; HPL, HAIC+PD-1 antibodies+lenvatinib; WBC, white blood cell; NE, neutrophil; Hgb, hemoglobin; PLT, platelet; ALB, albumin; ALT, alanine aminotransferase; PT, prothrombin; TBIL, total bilirubin; CRE, Creatinine; AFP, alpha-fetoprotein; HAIC, hepatic arterial infusion chemotherapy.

### Efficacy analysis

**Figure 2:**
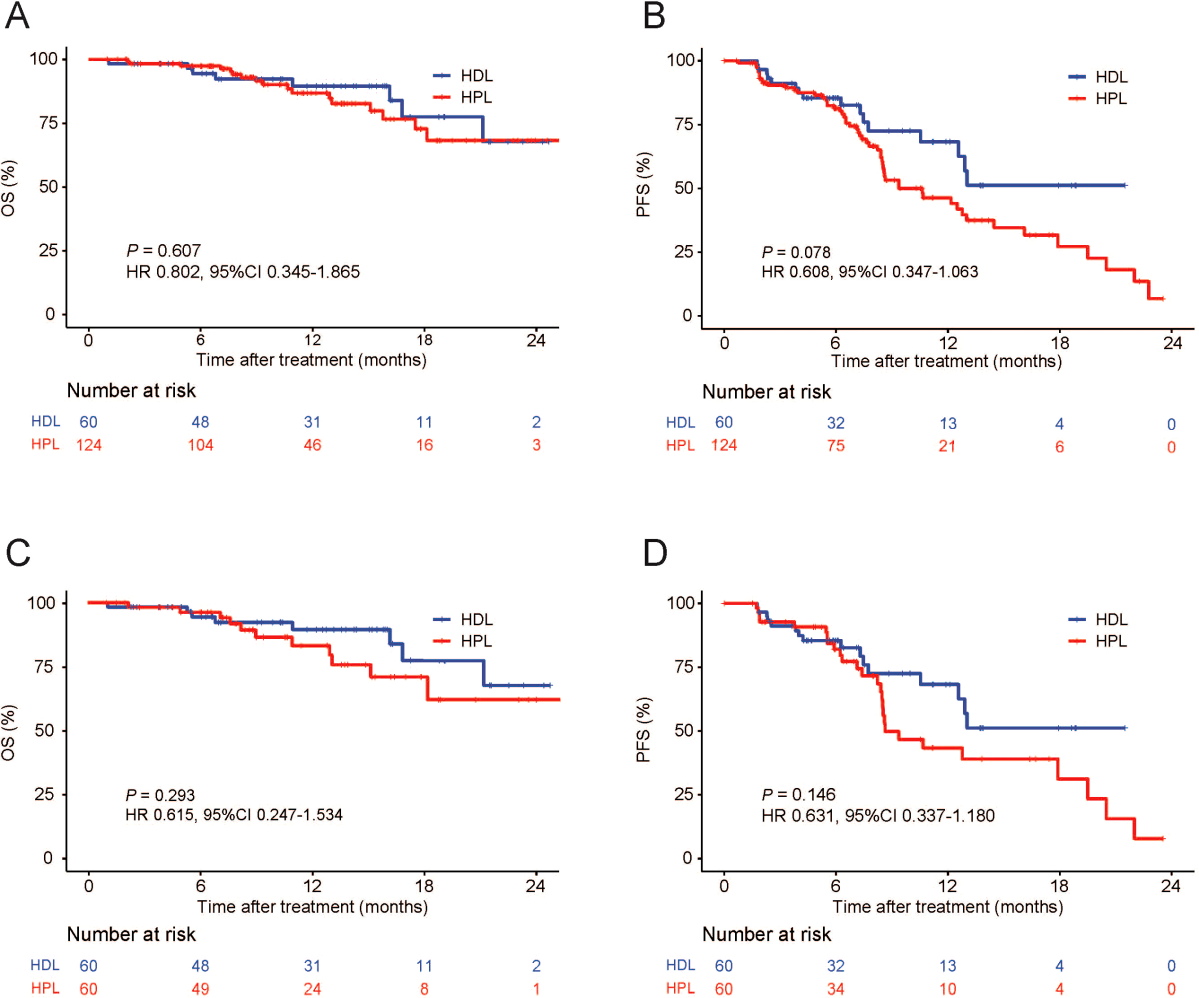
Kaplan–Meier curves for OS and PFS. Primary cohort **(A, B)**, PSM cohort **(C, D)**. OS, overall survival; PFS, progression-free survival; PSM, propensity score matching; HDL, HAIC+durvalumab+lenvatinib; HPL, HAIC+PD-1 antibodies+lenvatinib.

**Figure 3:**
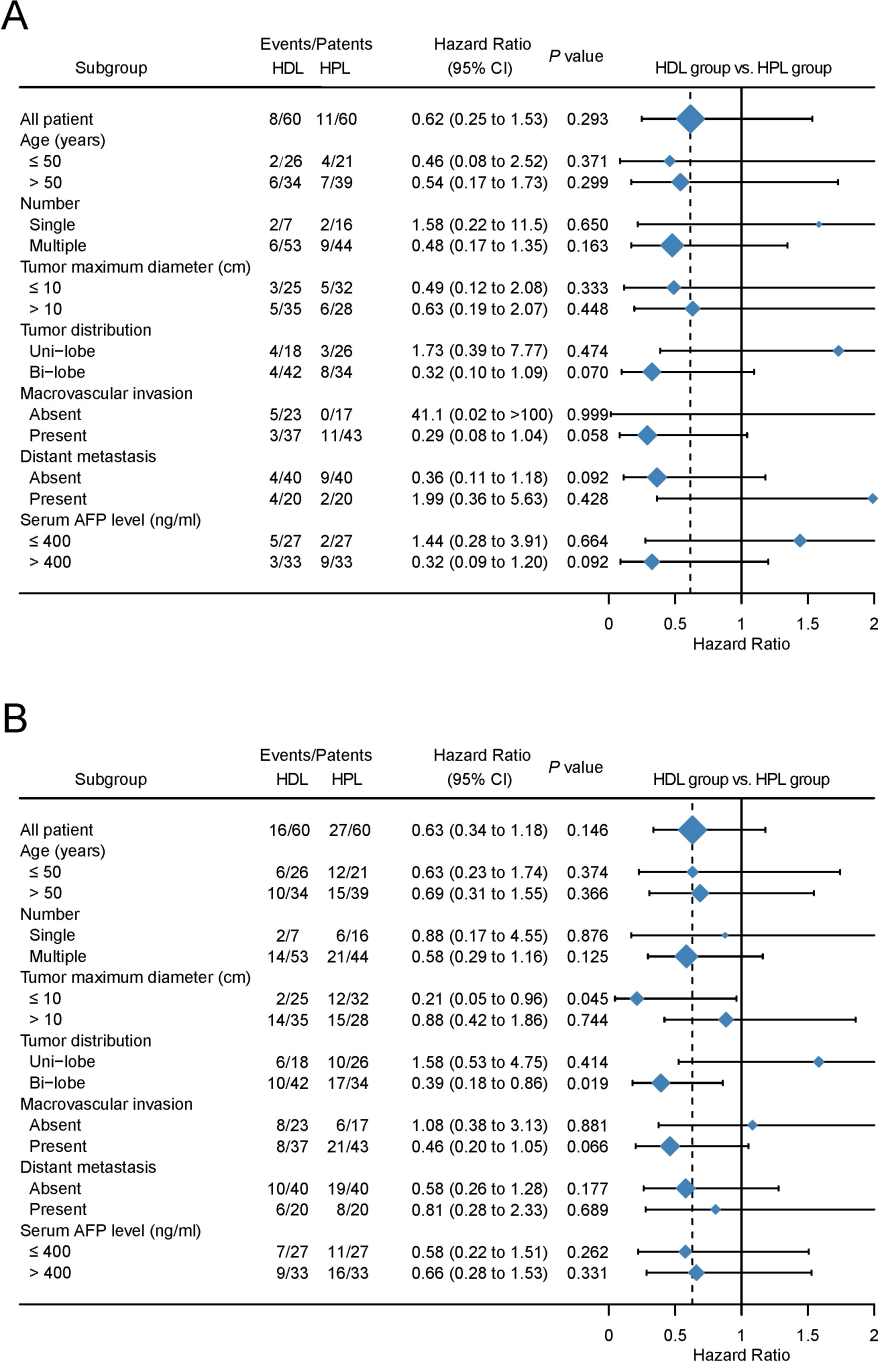
Forest plots by subgroup for OS **(A)** and PFS **(B)**.

The median OS and PFS are not evaluated on the data cut-off date of Match 10, 2023. The provided data illustrates the outcomes of two treatment groups, HDL and HPL, in terms of overall survival (OS) and progression-free survival (PFS) rates at various time intervals. For the HDL group, the OS rates at 6, 12, and 18 months were 94.5%, 89.5%, and 77.5%, respectively. In comparison, the HPL group exhibited OS rates of 97.4%, 86.8%, and 72.8% at the same intervals. Statistical analysis indicated no significant difference in OS between the two groups (p=0.607). In terms of PFS, the HDL group showed rates of 85.4%, 68.2%, and 51.2% at 6, 12, and 18 months, respectively. Contrastingly, the PFS rates for the HPL group were 81.4%, 46.3%, and 27.2% at the corresponding time points. Although the PFS rate of the HDL group suggested a trend of superiority over the HPL group, this difference was not statistically significant (p=0.078) (**Figures 2A, B**). Following PSM, the OS rates at 6, 12, and 18 months in the HDL group were 94.5%, 89.5%, and 77.5%, respectively, while those at 6, 12, and 18 months in the HPL group were 96.3%, 83.2%, and 71.1%, respectively, with no significant difference (p=0.293). The PFS rates at 6, 12, and 18 months in the HDL group were 85.4%, 68.2%, and 51.2%, respectively. The PFS rates at 6, 12, and 18 months in the HPL group were 82.1%, 43.4%, and 31.2%, respectively. There was no significant difference between the two groups (p=0.146) (**Figures 2C, D**). Subgroup analysis, as depicted in **Figures 3A, B**, highlighted similar OS benefits between the HDL and HPL groups across different subgroups. However, PFS benefits favored the HDL group in tumors with a maximum diameter ≤10 cm and involving both liver lobes.

**Figure 4:**
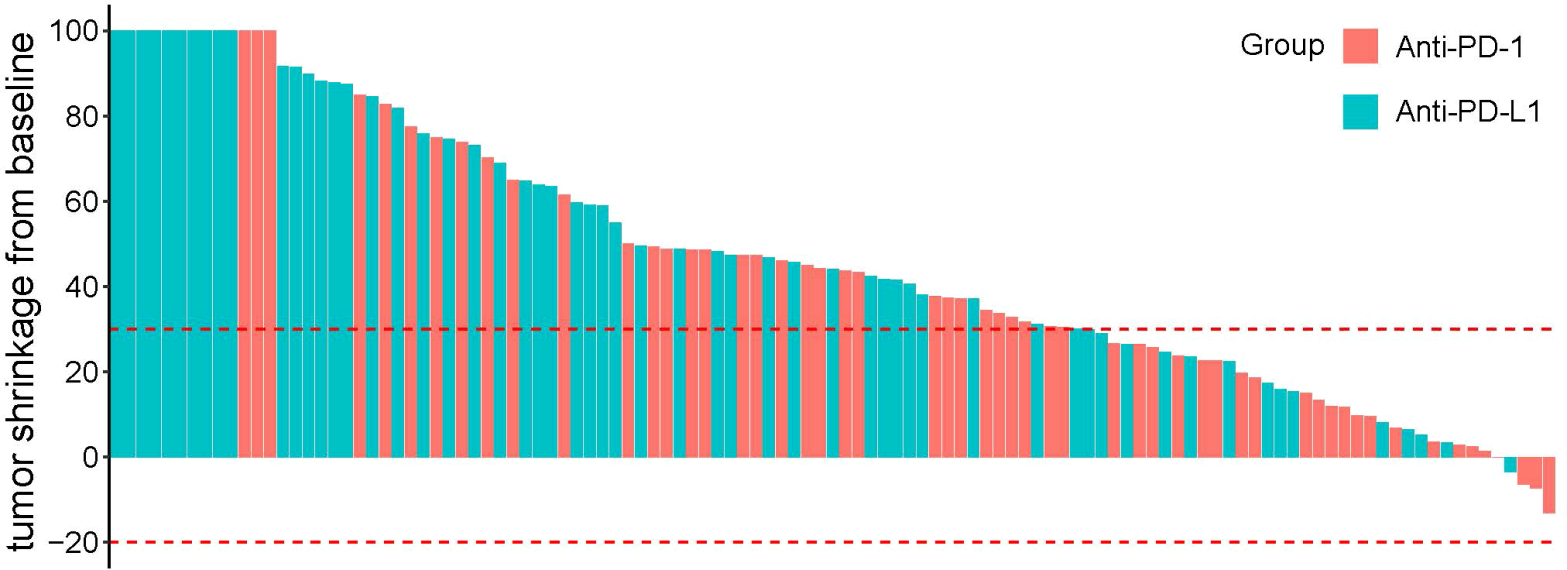
Chart of individual tumor response.

The treatment response is summarized in **Table 3**. In matching paired population, the CR rate (17.2%) of the HDL group was still significantly higher than HPL group (5.4%, p=0.046), and the ORR was 74.1%, which was also significantly higher than that of the HPL group (53.6%, p=0.022) according to mRECIST; and according to RECIST 1.1, ORR in the HDL group (60.3%) was significantly better than the 41.1% in the HPL group (p=0.040) as well. The individual tumor response is shown in **Figure 4**.

**Table 3:** Tumor response in patients in the HDL group and the HPL group after PSM.

	HDL group (n=58)	HPL group (n=56)	*P* value
mRECIST			
CR	10 (17.2%)	3 (5.4%)	0.046
PR	33 (56.9%)	27 (48.2%)	0.353
SD	11 (19.0%)	23 (41.1%)	0.010
PD	4 (6.9%)	3 (5.4%)	0.732
ORR	43 (74.1%)	30 (53.6%)	0.022
DCR	54 (93.1%)	53 (94.6%)	0.732
RECIST 1.1			
CR	0 (0.0%)	0 (0.0%)	1.000
PR	35 (60.3%)	23 (41.1%)	0.040
SD	19 (32.8%)	30 (53.6%)	0.025
PD	4 (6.9%)	3 (5.4%)	0.732
ORR	35 (60.3%)	23 (41.1%)	0.040
DCR	54 (93.1%)	53 (94.6%)	0.732

PSM, propensity score matching; HDL, HAIC+durvalumab+ lenvatinib; HPL, HAIC+PD-1 antibodies+lenvatinib; mRECIST, modified response evaluation criteria in solid tumors; RECIST, response evaluation criteria in solid tumors.

### Safety analysis

All adverse events (AEs) are listed in **Table 4**. The most common AEs happened were pain (48.33%), ALT level elevated (40%), Thrombocytopenia (30%), and anemia (23.33%) in the HDL group, and pain (41.67%), ALT level elevated (35%), vomiting (33.33%), and anemia (30%) in HPL group. The incidence rates of all grades of AEs were similar in the two groups, 93.3% in the HDL group and 96.67% in the HPL group (p=0.679). The HPL group exhibited a reduced proportion of patients experiencing Grade 1-2 anorexia (8.3% vs. 28.3%, p=0.008) and Grade 1-2 hyperbilirubinemia (1.7% vs. 16.7%, p=0.008) compared to the HDL group. The incidence of Grade ≥3 AEs was lower in the HDL group at 10% compared to 18.3% in the HPL group, although this difference was not statistically significant (p= 0.191).

**Table 4:** Summary of treatment-related adverse events.

Adverse Events,N(%)		HDL group (n=60)	HPL group (n=60)	p-value
Overall	All grades	56 (93.3)	58 (96.7)	0.679
	≥3 grade	6 (10.0)	11 (18.3)	0.191
ALT level elevated	All grades	24 (40.0)	21 (35.0)	0.706
	≥3 grade	0 (0.0)	0 (0.0)	NA
Anorexia	All grades	5 (8.3)	17 (28.3)	0.008
	≥3 grade	0 (0.0)	0 (0.0)	NA
Diarrhea	All grades	11 (18.3)	10 (16.7)	1.000
	≥3 grade	0 (0.0)	0 (0.0)	NA
Anemia	All grades	14 (23.3)	18 (30.0)	0.536
	≥3 grade	0 (0.0)	0 (0.0)	NA
Constipation	All grades	9 (15.0)	5 (8.3)	0.394
	≥3 grade	0 (0.0)	0 (0.0)	NA
Pain	All grades	29 (48.3)	25 (41.7)	0.582
	≥3 grade	0 (0.0)	1 (1.6)	1.000
Vomiting	All grades	11 (18.3)	20 (33.3)	0.094
	≥3 grade	0 (0.0)	1 (1.6)	1.000
Rash	All grades	8 (13.3)	4 (6.7)	0.362
	≥3 grade	2 (3.3)	0 (0.0)	0.496
Leukocytopenia	All grades	7 (11.7)	14 (23.3)	0.148
	≥3 grade	0 (0.0)	3 (5.0)	0.244
Neutropenia	All grades	10 (16.7)	17 (28.3)	0.189
	≥3 grade	1 (1.7)	7 (11.7)	0.061
Hypertension	All grades	7 (11.7)	8 (13.3)	1.000
	≥3 grade	0 (0.0)	1 (1.7)	1.000
Hypoalbuminemia	All grades	11 (18.3)	7 (11.7)	0.444
	≥3 grade	0 (0.0)	0 (0.0)	NA
Thrombocytopenia	All grades	18 (30.0)	14 (23.3)	0.536
	≥3 grade	2 (3.3)	0 (0.0)	0.496
Edema	All grades	3 (5.0)	4 (6.7)	1.000
	≥3 grade	0 (0.0)	0 (0.0)	NA
PT prolong	All grades	1 (1.7)	0 (0.0)	1.000
	≥3 grade	0 (0.0)	0 (0.0)	NA
Fever	All grades	2 (3.3)	7 (11.7)	0.163
	≥3 grade	0 (0.0)	0 (0.0)	NA
Cough	All grades	2 (3.3)	1 (1.7)	1.000
	≥3 grade	0 (0.0)	0 (0.0)	NA
Dysuria	All grades	3 (5.0)	4 (6.7)	1.000
	≥3 grade	0 (0.0)	0 (0.0)	NA
Insomnia	All grades	2 (3.3)	2 (3.3)	1.000
	≥3 grade	0 (0.0)	0 (0.0)	NA
pulmonary embolism	All grades	1 (1.7)	0 (0.0)	1.000
	≥3 grade	1 (1.7)	0 (0.0)	1.000
Hyperbilirubinemia	All grades	1 (1.7)	10 (16.7)	0.008
	≥3 grade	0 (0.0)	1 (1.7)	1.000
GI bleeding	All grades	0 (0.0)	1 (1.7)	1.000
	≥3 grade	0 (0.0)	1 (1.7)	1.000
Arrhythmia	All grades	0 (0.0)	1 (1.7)	1.000
	≥3 grade	0 (0.0)	0 (0.0)	NA
Infection	All grades	0 (0.0)	3 (5.0)	0.244
	≥3 grade	0 (0.0)	1 (1.7)	1.000
perianal abscess	All grades	0 (0.0)	1 (1.7)	1.000
	≥3 grade	0 (0.0)	1 (1.7)	1.000

HDL, HAIC+durvalumab+lenvatinib; HPL, HAIC+PD-1 antibodies+lenvatinib; ALT, alanine aminotransferase; GI, gastrointestinal; NA, not applicable.

## Discussion

The combination of local therapies with systemic treatments such as immunotherapy and targeted therapy has become a common approach in treating advanced liver cancer. This strategy aims to provide both localized control of the tumor and systemic disease management. Studies have suggested that HAIC may offer advantages over transarterial chemoembolization (TACE) as a local treatment for advanced liver cancer patients (16, 17). Additionally, our research group’s findings indicate that combining HAIC with lenvatinib and immunotherapy yields better efficacy compared to using lenvatinib and immunotherapy alone (6). The selection between PD-1 and PD-L1 inhibitors for immunotherapy has become crucial for clinicians. Understanding the differences in efficacy and safety profiles between these agents is essential for optimizing treatment outcomes.

In this study, The baseline is equilibrium between HDL and HPL groups after matching. In this retrospective study, the baseline clinical characteristics of patients showed a heavy tumor burden, with a maximum diameter of tumor in the HDL group of 10.55 (3.4-19.3) cm and in the HPL group of 9.95 (2.0-22.1) cm, with macroscopic invasion in more than 60% of patients and distance metastasis in more than 30% of patients. After PSM, There was no significant difference in OS rates between the HDL and HPL groups at 6, 12, and 18 months. After PSM, the PFS rates at 6, 12, and 18 months in the HDL group were 85.4%, 68.2%, and 51.2%, respectively. The PFS rates at 6, 12, and 18 months in the HPL group were 82.1%, 43.4%, and 31.2%, respectively, The PFS rate of the HDL group showed a trend of being superior to the HPL group, but there was still no statistically significant difference. We speculate that the reason why the benefit trend of PFS has not been translated into the benefit of OS may be due to the blurring of this difference by posterior treatment. The subgroup analysis of OS and PFS showed that the OS benefits of the two groups were similar, while the PFS benefits the HDL group in the tumors with a maximum diameter ≤ 10 cm and involving both livers. The lack of translation of PFS benefits into OS benefits could be attributed to subsequent treatments received by patients after the initial therapy, which might have confounded the survival outcomes. Subgroup analysis revealed similar OS benefits between the two treatment groups. However, the HDL group showed improved PFS in tumors with a maximum diameter of ≤10 cm and involving both liver lobes.

Results showed the ORR was higher in the HDL group than in the HPL group according to modified RECIST (mRECIST) (74.1% vs. 53.6%, p = 0.022) and RECIST 1.1 (60.3% vs. 41.1%, p = 0.040) respectively. The results of the HPL group ORR and retrospective study report on PD-1 combined with lenvatinib and HAIC in the treatment of HCC are similar (18). Harvard University’s Manish J. Butte et al. found that in addition to binding to PD-1, PD-L1 can also bind to B7.1 molecules (19). B7.1 is a co-stimulatory molecule expressed on the surface of antigen-presenting dendritic cells, which can bind to CD28 molecules on T cells to activate them. Dendritic cells inherently express PD-L1 molecules and can interact with their own B7.1 (20). PD-L1 monoclonal antibody may actually rescue these dendritic cells that are inhibited by PD-L1. This suggests that PD-L1 may have a higher ORR in anti-tumor therapy.

PD-L1 inhibitors are noted for their ability to preserve immune balance by not blocking PD-L2. This characteristic reduces the risk of severe immune-related adverse events (irAEs) (21). A meta-analysis of non-small cell lung cancer (NSCLC) patient data suggests that compared to PD-L1 inhibitor treatment, PD-1 inhibitor treatment may increase the incidence of irAEs, both in terms of any grade and high-grade (3-4 grades) irAEs (22). Durvalumab, an engineered Ig1 antibody, does not induce antibody-dependent cell-mediated cytotoxicity (ADCC) effects (23). In the safety analysis mentioned, it was observed that the incidence of any level of hyperbilirubinemia and anorexia was lower in the HDL group compared to the HPL group.

The results of this study indicate that triple therapy with PD-L1 yields significantly better tumor reduction effects than triple therapy with PD-1, and it also has a lower incidence of severe adverse events (AEs). Further prospective studies involving a larger patient population are necessary. Given the promising efficacy and safety of HAIC combined with lenvatinib and PD-L1 in clinical practice, our group initiated a prospective study (HDL-001, NCT04961918) to further evaluate this treatment regimen and address the remaining questions from this paper.

## Conclusion

In conclusion, PD-L1 antibody seems to be a better companion in the combination therapy of HAIC+ICIs+lenvatinib than PD-1 antibody for higher ORR and lower incidence of severe AEs. Further prospective study involving a larger population of patients is required.

## Data Availability

The original contributions presented in the study are included in the article/**Supplementary Material**. Further inquiries can be directed to the corresponding authors.

## References

[1] Bray F Laversanne M Sung H Ferlay J Siegel RL Soerjomataram I. Global cancer statistics 2022: GLOBOCAN estimates of incidence and mortality worldwide for 36 cancers in 185 countries. CA Cancer J Clin. (2024) 74:229–63. doi: 10.3322/caac.21834 38572751

[2] Han B Zheng R Zeng H Wang S Sun K Chen R. Cancer incidence and mortality in China, 2022. J Natl Cancer Cent. (2024) 4:47–53. doi: 10.1016/j.jncc.2024.01.006 39036382 PMC11256708

[3] Allemani C Matsuda T Di Carlo V Harewood R Matz M Niksic M. Global surveillance of trends in cancer survival 2000-14 (CONCORD-3): analysis of individual records for 37 513 025 patients diagnosed with one of 18 cancers from 322 population-based registries in 71 countries. Lancet. (2018) 391:1023–75. doi: 10.1016/S0140-6736(17)33326-3 PMC5879496 29395269

[4] Finn RS Qin S Ikeda M Galle PR Ducreux M Kim TY. Atezolizumab plus bevacizumab in unresectable hepatocellular carcinoma. N Engl J Med. (2020) 382:1894–905. doi: 10.1056/NEJMoa1915745 32402160

[5] Ren Z Xu J Bai Y Xu A Cang S Du C. Sintilimab plus a bevacizumab biosimilar (IBI305) versus sorafenib in unresectable hepatocellular carcinoma (ORIENT-32): a randomised, open-label, phase 2-3 study. Lancet Oncol. (2021) 22:977–90. doi: 10.1016/S1470-2045(21)00252-7 34143971

[6] Mei J Tang YH Wei W Shi M Zheng L Li SH. Hepatic arterial infusion chemotherapy combined with PD-1 inhibitors plus lenvatinib versus PD-1 inhibitors plus lenvatinib for advanced hepatocellular carcinoma. Front Oncol. (2021) 11:618206. doi: 10.3389/fonc.2021.618206 33718175 PMC7947809

[7] Abou-Alfa GK Lau G Kudo M Chan SL Kelley RK Furuse J. Tremelimumab plus durvalumab in unresectable hepatocellular carcinoma. NEJM Evid. (2022) 1:EVIDoa2100070. doi: 10.1056/EVIDoa2100070 38319892

[8] Lau G Abou-Alfa GK Cheng AL Sukeepaisarnjaroen W Dao TV Kang YK. Outcomes in the Asian subgroup of the phase III randomised HIMALAYA study of tremelimumab plus durvalumab in unresectable hepatocellular carcinoma. J Hepatol. (2024). doi: 10.1016/j.jhep.2024.07.017 39089633

[9] Shiina S Gani RA Yokosuka O Maruyama H Nagamatsu H Payawal DA. APASL practical recommendations for the management of hepatocellular carcinoma in the era of COVID-19. Hepatol Int. (2020) 14:920–9. doi: 10.1007/s12072-020-10103-4 PMC7655459 33174159

[10] He M Li Q Zou R Shen J Fang W Tan G. Sorafenib plus hepatic arterial infusion of oxaliplatin, fluorouracil, and leucovorin vs sorafenib alone for hepatocellular carcinoma with portal vein invasion: A randomized clinical trial. JAMA Oncol. (2019) 5:953–60. doi: 10.1001/jamaoncol.2019.0250 PMC6512278 31070690

[11] Li S Mei J Wang Q Guo Z Lu L Ling Y. Postoperative adjuvant transarterial infusion chemotherapy with FOLFOX could improve outcomes of hepatocellular carcinoma patients with microvascular invasion: A preliminary report of a phase III, randomized controlled clinical trial. Ann Surg Oncol. (2020) 27:5183–90. doi: 10.1245/s10434-020-08601-8 32418078

[12] Wolchok JD Hoos A O'day S Weber JS Hamid O Lebbe C. Guidelines for the evaluation of immune therapy activity in solid tumors: immune-related response criteria. Clin Cancer Res. (2009) 15:7412–20. doi: 10.1158/1078-0432.CCR-09-1624 19934295

[13] Lencioni R Llovet JM. Modified RECIST (mRECIST) assessment for hepatocellular carcinoma. Semin Liver Dis. (2010) 30:52–60. doi: 10.1055/s-0030-1247132 20175033 PMC12268942

[14] Eisenhauer EA Therasse P Bogaerts J Schwartz LH Sargent D Ford R. New response evaluation criteria in solid tumours: revised RECIST guideline (version 1.1). . Eur J Cancer. (2009) 45:228–47. doi: 10.1016/j.ejca.2008.10.026 19097774

[15] Chen JW Maldonado DR Kowalski BL Miecznikowski KB Kyin C Gornbein JA. Best practice guidelines for propensity score methods in medical research: consideration on theory, implementation, and reporting. A review. Arthroscopy. (2022) 38:632–42. doi: 10.1016/j.arthro.2021.06.037 34547404

[16] Li S Mei J Wang Q Shi F Liu H Zhao M. Transarterial infusion chemotherapy with FOLFOX for advanced hepatocellular carcinoma: a multi-center propensity score matched analysis of real-world practice. Hepatobiliary Surg Nutr. (2021) 10:631–45. doi: 10.21037/hbsn PMC8527433 34760967

[17] Li QJ He MK Chen HW Fang WQ Zhou YM Xu L. Hepatic arterial infusion of oxaliplatin, fluorouracil, and leucovorin versus transarterial chemoembolization for large hepatocellular carcinoma: A randomized phase III trial. J Clin Oncol. (2022) 40:150–60. doi: 10.1200/JCO.21.00608 34648352

[18] Cao G Wang X Chen H Gao S Guo J Liu P. Hepatic arterial infusion chemotherapy plus regorafenib in advanced colorectal cancer: a real-world retrospective study. BMC Gastroenterol. (2022) 22:328. doi: 10.1186/s12876-022-02344-4 35788189 PMC9251591

[19] Butte MJ Keir ME Phamduy TB Sharpe AH Freeman GJ. Programmed death-1 ligand 1 interacts specifically with the B7-1 costimulatory molecule to inhibit T cell responses. Immunity. (2007) 27:111–22. doi: 10.1016/j.immuni.2007.05.016 PMC2707944 17629517

[20] Chaudhri A Xiao Y Klee AN Wang X Zhu B Freeman GJ. PD-L1 binds to B7-1 only in cis on the same cell surface. Cancer Immunol Res. (2018) 6:921–9. doi: 10.1158/2326-6066.CIR-17-0316 PMC7394266 29871885

[21] Chen DS Irving BA Hodi FS. Molecular pathways: next-generation immunotherapy–inhibiting programmed death-ligand 1 and programmed death-1. Clin Cancer Res. (2012) 18:6580–7. doi: 10.1158/1078-0432.CCR-12-1362 23087408

[22] Sun X Roudi R Dai T Chen S Fan B Li H. Immune-related adverse events associated with programmed cell death protein-1 and programmed cell death ligand 1 inhibitors for non-small cell lung cancer: a PRISMA systematic review and meta-analysis. BMC Cancer. (2019) 19:558. doi: 10.1186/s12885-019-5701-6 31182061 PMC6558759

[23] Stewart R Morrow M Hammond SA Mulgrew K Marcus D Poon E. Identification and characterization of MEDI4736, an antagonistic anti-PD-L1 monoclonal antibody. Cancer Immunol Res. (2015) 3:1052–62. doi: 10.1158/2326-6066.CIR-14-0191 25943534

