# Supplementary material for: Comparing PD-L1 with PD-1 antibodies combined with lenvatinib and hepatic arterial infusion chemotherapy for unresectable hepatocellular carcinoma
Source: Front Immunol. 2024 Oct 23;15:1491857. doi: 10.3389/fimmu.2024.1491857 (PMC11537909; doi:10.3389/fimmu.2024.1491857)
Supplement: Supplementary file 1 [file Table1.docx]

| Category | Patients  Number (percent) |
| --- | --- |
| Tislelizumab (Baiji Shenzhou (Beijing) Biotechnology Co Ltd (Inst) | 30 (24.2%) |
| Sintilimab (Innovent Biologics (Suzhou, Jiangsu, China)) | 51 (41.1%) |
| Camrelizumab (Jiangsu Hengrui Pharmaceutical) | 7 (5.6%) |
| Toripalimab (Shanghai Junshi Bioscience) | 26 (21.0%) |
| Pembrolizumab (Merck Sharp & Dohme Corp) | 10 (8.1%) |

Supplementary Table 1: Proportion of PD-1 antibodies used in the HDL cohort and the HPL cohort.

Abbreviations: HDL, HAIC+durvalumab+lenvatinib; HPL, HAIC+PD-1 antibodies+lenvatinib
